# Exploring the behavioural drivers of veterinary surgeon antibiotic prescribing: a qualitative study of companion animal veterinary surgeons in the UK

**DOI:** 10.1186/s12917-018-1646-2

**Published:** 2018-11-07

**Authors:** C. King, M. Smith, K. Currie, A. Dickson, F. Smith, M. Davis, P. Flowers

**Affiliations:** 1 Department of Nursing and Community Health, School of Health and Life Sciences, Glasgow Caledonian University, Cowcaddens Road, Glasgow, G4 0BA Scotland, UK; 2 School of Social Sciences, Monash University, Melbourne, Australia

**Keywords:** Antibiotics, Antimicrobial resistance, AMR, Antimicrobial stewardship, AMS, Prescribing behaviours, Companion animals, Veterinary surgeons, Qualitative

## Abstract

**Background:**

Multi-drug resistant bacteria are an increasing concern in both human and veterinary medicine. Inappropriate prescribing and use of antibiotics within veterinary medicine may be a contributory factor to antimicrobial resistance (AMR). The ‘One Health’ Initiative aims to work across species and environments to reduce AMR, however; little is currently known about the factors which influence antibiotic prescribing among veterinary surgeons in companion animal practice.

This paper reports on qualitative data analysis of interviews with veterinary surgeons whose practice partially or wholly focuses on companion animals (*N* = 16). The objective of the research was to explore the drivers of companion animal veterinary surgeons’ antibiotic prescribing behaviours. The veterinary surgeons interviewed were all practising within the UK (England (*n* = 4), Scotland (*n* = 11), Northern Ireland (*n* = 1)). A behavioural thematic analysis of the data was undertaken, which identified barriers and facilitators to specific prescribing-related behaviours.

**Results:**

Five components of prescribing behaviours were identified: 1) confirming clinical need for antibiotics; 2) responding to clients; 3) confirming diagnosis; 4) determining dose, duration and type of antibiotic; and 5) preventing infection around surgery (with attendant appropriate and inappropriate antibiotic prescribing behaviours). Barriers to appropriate prescribing identified include: business, diagnostic, fear, habitual practice and pharmaceutical factors. Facilitators include: AMR awareness, infection prevention, professional learning and regulation and government factors.

**Conclusion:**

This paper uses a behavioural lens to examine drivers which are an influence on veterinary surgeons’ prescribing behaviours. The paper contributes new understandings about factors which influence antibiotic prescribing behaviours among companion animal veterinary surgeons. This analysis provides evidence to inform future interventions, which are focused on changing prescribing behaviours, in order to address the pressing public health concern of AMR.

## Background

Inappropriate antibiotic use in companion animals (dogs, cats, rabbits that live in households) has been identified as potentially contributing to antimicrobial resistance (AMR) [1]. The extent of the contribution of antibiotic use within companion animal populations to AMR remains unknown. In the UK alone, it is estimated that 45% of all households have a pet with 26% of households in the UK owning a dog and 18% of households owning a cat [2]. While antibiotic prescribing for companion animals has been decreasing in recent years [1], there remains substantial potential for inappropriate antibiotic use in the companion animal population to drive AMR. The proximity of humans and companion animals in domestic environments means that there also is potential for the transfer of antibiotic resistant bacteria between species [3, 4]. This inter-species transmission of resistant bacteria has implications for both human and animal health [5].

The complexity of the relationships between AMR, human and animal health requires an inter-disciplinary approach that allows for the combination of a range of research methods and expertise. Until now, the focus within veterinary practice has been on identifying the reasons why antibiotics may be used indiscriminately in large-animal populations, for example, cattle and sheep [6]. To our knowledge, our study is the first UK-based AMR research to use a behavioural lens to analyse qualitative accounts of companion animal veterinary surgeons in order to understand antibiotic prescribing behaviours. A focus on understanding the behavioural components of prescribing is particularly relevant and useful in the context of AMR and antimicrobial stewardship (AMS) as it provides evidence for the development of behavioural interventions [7]. These interventions can provide policy-makers and practitioners with practical, focused and evidenced ways to implement AMS programmes aimed at reducing AMR. The behavioural approach taken in this study builds on a developing body of literature using qualitative methods to explore the drivers of antibiotic use in small animal veterinary practice [8]. It explores both the broad behavioural domain of prescribing (the clustering of specific behaviours that relate to prescribing) and the determinants, or antecedents, of such behaviours.

### Research aim and questions

The aim of this study was to identify key companion animal veterinary antibiotic prescribing behaviours, which might be amenable to change, in order to reduce drivers of AMR.

### Research questions

1. What are the component behaviours related to companion animal veterinary surgeon antibiotic prescribing?
2. What are the barriers and facilitators of appropriate antibiotic prescribing by companion animal veterinary surgeons?

## Methods

The study was approved by the Ethics Committee at Glasgow Caledonian University (Reference: HLS/NCH/16/001). Participating veterinary surgeons were recruited voluntarily using email invitations circulated through professional networks and connections from Health Protection Scotland (HPS) and the Control of Antimicrobial Resistance Scotland (CARS) project steering group. Written information was provided and informed consent was obtained prior to interview.

Individual telephone or face-to-face interviews were conducted at a time convenient to the participant and lasted between 25 and 40 min. Three experienced members of the research team carried out the interviews (MS, FS, MD). Individual researcher’s skills have been developed through advanced methods training, previous research, and interview and analysis practicums held by the Principal Investigator of the research, who is a leader in the field [9].

The semi-structured interview schedule was designed to provide a framework for the veterinary surgeons to express their views and experiences of antibiotic prescribing and resistance freely and in depth. Interview topics, with related questions, included: exploring veterinary surgeons use of antibiotics in practice; influences on their prescribing of antibiotics; understandings of AMR and AMS; barriers and facilitators of AMS; challenges and facilitators to using guidance for AMS; and their future use of antibiotics. In keeping with the exploratory, inductive focus of the research no a priori behavioural categories were imposed at this stage of the research to give the interviewees opportunity to respond freely and so that the interviewers could follow-up on important emergent themes.

All interviews were recorded and transcribed then imported into NVivo (Version 10) (Computer Software for Qualitative Data Analysis). Principles of thematic analysis (Braun & Clark 2006) [10] were applied to identify components of prescribing behaviour and to categorise associated barriers and facilitators to each aspect of the behaviour. The components of antibiotic prescribing behaviour outlined in Table 1 were generated through a two stage process: first, all behaviours relating to AMR and AMS were identified. Three key behaviours were identified: prescribing; the use of diagnostics; and interactions between veterinary surgeons and pet-owners (reported in Smith et al., 2018) [11]. Second, the overall category of prescribing behaviour was analysed further to identify distinct component behaviours. While it is recognised that these component behaviours overlap the detailing of the five different components was felt by the research team to be useful in fully understanding the behaviour.

**Table 1:** Components of antibiotic prescribing behaviour

Component behaviour	Appropriate behaviour	Inappropriate behaviour
1. Confirming clinical need for antibiotic	Identified clinical need for antibiotic	Cautionary prescribing ‘just in case’ antibiotics are required
2. Responding to clients	Providing client education on antibiotic use	Responding to perceived client pressure
3. Confirming diagnosis	Use of diagnostic tests to confirm antibiotic need	Prescribing antibiotics without confirmed diagnosis
4. Dose, duration and type of antibiotic	Accurate prescribing: dose and duration of antibiotic use in line with guidelines	Inaccurate prescribing: prescribing too high or too low a dose of antibiotics or too short or long a course of antibiotics or the wrong type of antibiotic
5. Preventing infection around surgical interventions	Enhanced infection prevention and control measures around surgery	Prescribing antibiotics as a preventative measure related to surgical interventions

Rigour in analysis was ensured by peer review within the research team. This process involved initial coding of the data to identify a coding map (CK) which was reviewed independently by two other members of the research team (MS and PF). All three researchers then discussed the coding map, negotiated discrepancies and clarified the meanings of all the codes. The coded behaviours were then tabulated alongside their barriers and facilitators and reviewed by the full research team.

## Results

Sixteen companion animal veterinary surgeons from across the UK (Scotland, England, Northern Ireland) took part in interviews. Areas of practice ranged from front-line services, consulting directly with members of the public, to secondary referral services in specialist centres. Nine of the participants were male and seven were female. Eleven of the veterinary surgeons were currently based and working in Scotland, four in England and one in Northern Ireland.

Within this paper we explore the behaviour of companion animal veterinary surgeons’ antibiotic prescribing. First, we identify five specific components within prescribing-related behaviour. Second, we examine the factors that can act as either barriers or facilitators to these specific prescribing behaviours.

### Understanding prescribing behaviour

In Table 1 the five distinct yet overlapping components of antibiotic prescribing behaviour that led to either appropriate or inappropriate prescribing in specific contexts are described.

### Barriers and facilitators to appropriate prescribing behaviours

**Fig. 1:**
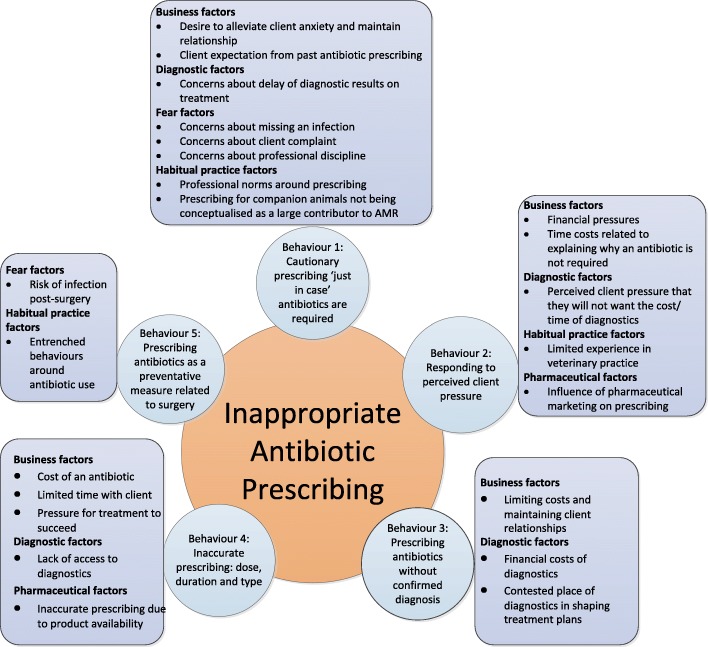
Barriers to appropriate antibiotic prescribing

**Fig. 2:**
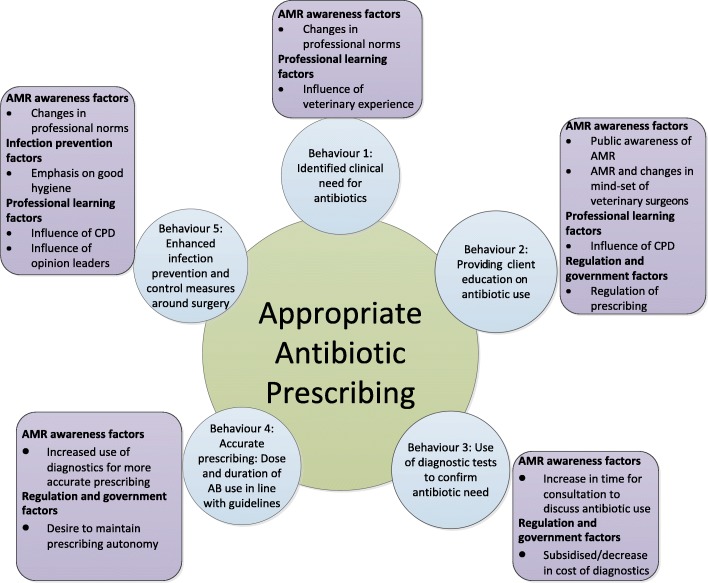
Facilitators to appropriate antibiotic prescribing

Veterinary surgeons’ accounts suggest that prescribing is influenced by a range of barriers and facilitators which have a ‘push-pull’ effect on prescribing behaviour. Five drivers were found to act as barriers to appropriate prescribing. The barriers were: business, diagnostic, fear, habitual practice and pharmaceutical factors. Four drivers were found to facilitate appropriate prescribing. The facilitators were: AMR awareness, infection prevention, professional learning and regulation and government factors. The key barriers and facilitators influencing appropriate prescribing behaviours are described below and summarised in Figs. 1 and 2.

Barriers to appropriate prescribing:

### Business factors

Veterinary surgeons talked about the tensions, which they experienced, between maintaining a viable business, client satisfaction and appropriate antibiotic prescribing:

*… people are our customers and they are what keeps the business going, so if we annoy them and there is another veterinary surgeon practice they can go to where they may just be handed out antibiotics [they will potentially do that] (Veterinary surgeon 1)*

Clients’ desires for their pet to recover could, at times, be in conflict with the appropriate prescribing of antibiotics. Antibiotics were often seen as direct action and symbolic of a clear pathway to a pets’ recovery compared to having to ‘wait it out’ while they recovered without medication:

*… owners don't want to say that [they want an antibiotic], but they want their animal better. … You want to make the animal better, therefore you think ‘Oh sod it, I’ll just give them a jag (Scottish version of the word injection)’. (Veterinary surgeon 13)*

Although veterinary surgeons themselves identified that it was increasingly rare for a client to ask directly for an antibiotic the implicit assumption was that they would want the quickest and most effective treatment. Client satisfaction was identified by veterinary surgeons as important for business success and, as such, could drive inappropriate prescribing behaviour.

### Diagnostic factors

The role of diagnostics in shaping treatment plans was contested in veterinary surgeons’ accounts. Almost all of the veterinary surgeons talked about not using diagnostics as much as they felt that they should:

*I will do culture and sensitivity but maybe nowhere near as often as I should. And I think most vets in the country would probably admit to that. (Veterinary surgeon 2)*

Despite an expressed desire by veterinary surgeons to use diagnostics more to inform their prescribing, the barriers of cost and time often influenced their decision-making around whether they would decide to use a diagnostic test:

*… the dog comes to the vet vaguely unwell and we can’t find out what’s wrong with it without spending money doing tests … or you could just give them a shot of antibiotics and see what it does (Veterinary surgeon 1)*

Diagnostics, then, held a contested place in veterinary surgeons’ accounts. They were recognised as an important facilitator of appropriate prescribing, yet their actual use in practice was inhibited by resource constraints.

### Fear factors

A key driver of inappropriate prescribing discussed by veterinary surgeons related to feelings of fear. Fear related to concerns that if they did not diagnose and treat an infection this could have negative consequences for an animal and for themselves, professionally. Although veterinary surgeons were aware of AMR, the fear of not diagnosing an infection could still act as an over-riding influence on their antibiotic prescribing behaviour:

*… fear, I think for the intra-operative, they’re frightened not to use them [antibiotics] in case they get infections (Veterinary surgeon 15)*

The fear of missing an infection, and potential professional consequences, were also magnified for veterinary surgeons with the forever present possibility of client complaint or disciplinary action through their professional bodies:

*… vets are completely paranoid the Royal Veterinary College [sic Royal College of Veterinary Surgeons] is going to cause them damage or get them struck off (Veterinary surgeon 5)*

As such, veterinary surgeons’ fears related to outcomes for companion animals and consequences for themselves professionally could act as barriers to appropriate prescribing.

### Habitual practice factors

Many of the veterinary surgeons talked about prescribing patterns which had been established over time and which influenced clients’ expectations of when their pet would receive an antibiotic. The examples of kennel cough and the treatment of cat abscesses were often used by veterinary surgeons to illustrate this point:

*There is some kind of pattern generated … this is what I've always treated this with, a jag (Scottish version of the word injection) of penicillin for a cat bite abscess. It's a hard habit to get out of. (Veterinary surgeon 2)*

Peer influence was viewed to be a powerful factor in shaping prescribing behaviours within veterinary surgeon practice:

*… the new grads are initially more prone to not give antibiotics because they were taught, well actually it’s bad, and they stand their ground more. But then as they get in to practice and get more experience and maybe they just get worn down or maybe the daily life … then they start giving antibiotics more loosely. (Veterinary surgeon 4)*

Veterinary surgeons also talked about witnessing what they felt to be inappropriate use of antibiotics and the conflict that this caused them:

*… as I say, I know certain individuals who will happily go and pick up fluoroquinolone before they would touch anything else. (Veterinary surgeon 13)*

*So like a skin infection, for example, should ideally have a three-week course … but in some cases we are discouraged from giving out what I would think would be an appropriate length of course, just because it will keep costs down. (Veterinary surgeon 3)*

As such, entrenched prescribing behaviours and the peer processes that could encourage these behaviours to develop, were barriers to appropriate prescribing.

### Pharmaceutical factors

Veterinary surgeons also identified that pharmaceutical companies influenced antibiotic prescribing. This opportunity to influence prescribing was created by the marketing of products to address challenges around the administration of antibiotics, such as, difficulties in getting cats to consume tablets. Veterinary surgeons, themselves, were conflicted about whether the use of these antibiotic products was likely to increase or decrease AMR. Irrespective of the nature of the impact on AMR, however, the influence of pharmaceutical companies on prescribing behaviours was articulated:

*… we do use [antibiotic injections] in cats and we know the problems with it, but we do it when we feel that the owners will not be able to give tablets … we prescribe it quite often to be honest. … I am not aware of much evidence that it contributes to specific antimicrobial resistance, but it is a third generation Cephalosporin … (Veterinary surgeon 11)*

As such, prescribing trends were influenced by internal and external factors. These factors included the prescribing norms of veterinary surgeons' practice and the influence of the pharmaceutical companies on prescribing.

Facilitators of appropriate prescribing:

### AMR awareness factors

Veterinary surgeons talked about how an increase in public awareness around AMR could help to shape clients’ expectations of antibiotic prescribing and use:

*The public are aware … there are certain things that aren't needing antibiotics that they thought they were generations ago, and that doctors are being a lot more tight with them. … they know it's an issue. (Veterinary surgeon 2)*

Many of the veterinary surgeons interviewed felt that they were often advocating for the appropriate use of antibiotics to colleagues who were less engaged with the issue of AMR. Over time, however, they found that they were able to influence colleagues to be more reflective of their prescribing behaviours:

*I mean we just used antibiotics willy-nilly, so I think it's only since a few of us have got interested in it, that it's actually brought it home to everyone that it's quite important what we do. (Veterinary surgeon 13)*

Increased awareness of AMR for both the public and within the veterinary surgeon profession was seen as an important driver of appropriate prescribing.

### Infection prevention factors

Emphasis on good hygiene was an important facilitator relating to prophylactic prescribing of antibiotics for surgery. Veterinary surgeons felt that, with good infection prevention practices in place, antibiotics are not necessary. Overall, it was felt by veterinary surgeons that there was a definite trend away from the prophylactic use of antibiotics for surgery:

*Prophylactically after surgery we do it very rarely, it's certainly not a routine thing … (Veterinary surgeon 12)*

Shifts in antibiotic prescribing relating to their prophylactic use were a useful example of how prescribing behaviours had changed over time and in relation to concerns about AMR.

### Professional learning factors

Patterns of antibiotic prescribing were established over time and linked closely to clients’ expectations around antibiotic use. However, veterinary surgeons talked about how these established behaviours could be mediated or changed with the correct professional education, experience and peer support:

*I say from 30 years ago till now, I think there is a vast improvement in awareness … younger generations that are coming out [from] the vet schools, it’s becoming almost part of just their mind-set. (Veterinary surgeon 15)*

Both graduate and post-graduate learning were talked about as important influences, along with the application of new knowledge in practice, peer support and willingness to change practices:

*… it gets easier as you get older and the clients respect you a bit more and your opinions. … if you can educate the more established vets in good ways, they're the ones that are teaching the younger vets and they're the ones who are teaching, in inverted commas, the clients what to expect. (Veterinary surgeon 2)*

In summary, learning from graduate education, experience of working as a practising veterinary surgeon and learning from peers were working in synergy with one another to create a positive climate of support for AMS, including appropriate prescribing.

### Regulation and government factors

Currently, veterinary surgeons in the UK can both prescribe and dispense antibiotics. The decoupling of prescription from the sale of antibiotics within veterinary practice would mean that veterinary surgeons could no longer dispense antibiotics to their clients. The potential for decoupling to happen in the UK was discussed by many of the veterinary surgeons. A lack of agreement about whether decoupling would facilitate AMS was evident. Some veterinary surgeons felt that it would improve AMS and others felt that it would be detrimental to animal welfare and act to limit veterinary surgeons’ professional autonomy. Irrespective of veterinary surgeons’ personal perspective on decoupling, however, there was agreement that appropriate prescribing was necessary. Improved prescribing could either be enabled by de-coupling or more appropriate prescribing could ward it off, as these two opposing viewpoints illustrate:

*I think that [decoupling] would be a good idea, because it would make us think very hard before handing them [antibiotics] out. As I say, they're so accessible for us that it's the easiest thing to do. (Veterinary surgeon 13)*

*… in the future we may not be able to prescribe them [antibiotics], or the prescription of them may be taken out of our hands, is the other possibility, that we don’t have the power to prescribe them and therefore we are completely stuffed then. (Veterinary surgeon 1)*

Governments were also seen to have a facilitative role in enabling AMS through the monitoring of antibiotic use and subsidising the cost of diagnostics:

*… thinking of government input, if they're going to start subsidising culture and sensitivity in labs, so the clients aren't really having to pay too much for it, that might make it a little bit easier. (Veterinary surgeon 2)*

As such, regulation and governmental input, although contested in terms of the form it should take, were talked about by veterinary surgeons as facilitative of appropriate prescribing.

## Discussion

The qualitative study reported in this paper takes a behavioural approach, providing new insights and ways of thinking about the contribution to AMR of the prescribing behaviours of companion animal veterinary surgeons. This study identified barriers and facilitators which drive the appropriate and inappropriate prescribing behaviours of companion animal veterinary surgeons.

The antibiotic prescribing behaviours of veterinary surgeons has now been the focus of a number of studies [6, 8, 12, 13], providing an important knowledge base to inform AMR-related behaviour change interventions. These studies use a range of research methodologies and show that antimicrobial prescribing among large-animals in the agricultural sector is influenced by a wide range of social, cultural and political factors [6], provide a pan-European perspective on veterinary surgeon antibiotic prescribing habits and use of sensitivity testing [12], explore the factors influencing prescribing in antibiotic dry cow therapy [13], and document factors influencing decision-making in antimicrobial prescribing in small animal veterinary practice in the UK [8].

With the exception of the work of Mateus and colleagues (2014) [8], the majority of the research to-date focuses primarily on food-producing animals with less attention paid to companion animal prescribing. Mateus et al. (2014) [8] explore influences on antimicrobial prescribing in relation to companion animals and highlight intrinsic factors relating to the veterinary surgeons, and extrinsic factors relating to the workplace and colleagues and to the pet owners and animals and their influence on decision-making. In the present study, we build on this research focusing primarily on the behavioural aspects of prescribing with the intent of developing the evidence-base on the drivers of AMR to inform future behavioural interventions aimed at AMS in the context of companion animal veterinary prescribing. Elsewhere we discuss factors associated with the interactions between veterinary surgeons and their clients [11]. In this paper, we focus on the factors driving the prescribing behaviours of the veterinary surgeons. In what follows, we reflect on our findings in light of the factors identified in Mateus et al.’s (2014) [8] study therefore building the field of knowledge about the promotion of AMS in companion animals.

Our research showed that business factors, including the importance of client retention were barriers to appropriate prescribing. Mateus et al. (2014) [8] report that business factors, although present in the accounts of veterinary surgeons, were not key drivers of decision-making. We found that veterinary surgeons were focused on maintaining good interactions with their clients to ensure the future of their veterinary business. Client expectations rarely related to a direct request for an antibiotic, but rather to veterinary surgeons’ perceptions that the costs of diagnostic tests would be too high and take too much time. Other studies have also shown that economic factors play an important role in shaping prescribing behaviours of veterinary surgeons, for example, in relation to veterinary surgeons’ awareness of farmers’ financial pressures and the intersection between antibiotic use as a short-term measure and more expensive but longer-term solutions to managing infections [6]. The identification of economic factors in studies carried out in different contexts suggests that interventions aimed at changing prescribing behaviours must recognise the economic context which influences veterinary surgeon prescribing.

Diagnostic factors, related to the cost and use of diagnostics, were a key barrier to appropriate prescribing. Mateus et al. (2014) [8] also highlighted factors related to diagnostic use as important influences on decision-making in antibiotic prescribing. Studies have shown that the rate of use of laboratory tests to support prescribing decisions can be very low [14–16]. Eschler and colleagues (2011) [14] found that only 3.7% cases of fluoroquinalone prescriptions for companion animals were supported with laboratory testing; and only around half of the respondents in Hughes and colleagues (2012) [15] survey of antimicrobial prescribing patterns in small animal practice identified laboratory tests to be an important influence on their antimicrobial decision-making. Although veterinary surgeons talk about laboratory tests as being a useful guide for appropriate prescribing, currently financial and time costs appear to be prohibitive factors in their actual use.

Fear factors, in particular in relation to the consequences of untreated infection, were identified as drivers of inappropriate prescribing. This type of cautionary prescribing appears to be context-dependent to companion animal practice. In studies exploring the barriers and facilitators to prescribing in other sectors such as pigs [6] and dairy cows [17], barriers tended to related more to managing the health and welfare of herds and groups of animals and the economic factors of farming and less to ensuring the survival of any one animal.

Habitual practice factors, related to veterinary surgeons’ opportunities to develop their knowledge and awareness of AMR and to change their prescribing behaviours, are key barriers to appropriate prescribing. Although trends in antibiotic prescribing are changing, the pace of behaviour change can be slow due to factors such as time pressures in veterinary surgeon practices and lack of access to continuing professional development. In Mateus et al.’s (2014) [8] study veterinary surgeons’ past experiences and habits were also found to be major contributors to their prescribing decisions. Behavioural interventions, which aim to change prescribing behaviours, need to acknowledge the importance of these factors.

Pharmaceutical factors were found to have a strong-hold on prescribing behaviours, which has been demonstrated in other studies. In one study of small animal veterinary practices [15], 70% of survey respondents reported that pharmaceutical companies were an important source of prescribing information.

AMR awareness and professional learning factors relating to a change in veterinary surgeons’ collective mind-set in relation to antibiotic prescribing was reported to facilitate appropriate prescribing. Although in Mateus et al’s (2014) [8] study peer pressure was not deemed to be a direct influence on which antibiotic was prescribed, our study suggests that peer influence may work through subtle and indirect mechanisms which could be beneficial for developing AMS interventions. As awareness of AMR increases, there are also opportunities for the use of techniques such as social comparison. This technique is where veterinary surgeons assess their stewardship performance in relation to that of others. Overall, both this and Mateus et al’s study [8] suggest that access to opportunities for continuing professional development and engagement with peers around AMR are likely to be important contributors to countering established habits and norms related to prescribing.

Infection prevention factors identified in this study as facilitative of appropriate prescribing are a useful example of how behaviours can and do change through the availability of alternatives to ensure animal health and welfare.

Regulation and government factors were identified as facilitative to appropriate prescribing, although there was not always agreement among veterinary surgeons about what form regulation should take. The identification of these facilitators suggests that the focus for behavioural change cannot be at the level of individual veterinary surgeons alone. Studies describing prescribing patterns of antimicrobial prescribing in small animal veterinary surgeon practice in the UK [1, 15, 18] and internationally [19] highlight that more antimicrobial prescribing guidelines, which encourage appropriate and more limited use, could be helpful and could act as a more tacit form of regulation.

### Limitations of the study

The study sample is limited to a self-selected group of veterinary surgeons with a professional interest in AMR and does not necessarily reflect the diversity of veterinary surgeons’ views. As such, it is likely that the veterinary surgeons who were interviewed would have been more progressive both in their ideal and actual behaviours relating to antibiotic prescribing than other veterinary surgeons who are less engaged with the AMR agenda. Although it is not possible to generalise the findings of this study, the authors believe that the value of the research is in describing the components of inappropriate prescribing behaviour, and the barriers and facilitators which drive these behaviours, in depth. Despite the limitation of the self-selected sample, the authors believe that the findings have relevance, for veterinary surgeons, and for all parties interested in a ‘One Health’ approach to tackling AMR.

### Future directions

This study was part of a larger programme of work focused on developing interventions to reduce AMR. It is intended that this data set will be combined with the other data sets of the study and using Theoretical Domains Framework [20, 21], the Behaviour Change Wheel [7] and the Behaviour Change Taxonomy [22] will be used to design AMS evidence-based behaviour change interventions.

## Conclusions

By applying a behavioural lens to the qualitative analysis reported in the findings of this paper, we have been able to identify specific antibiotic prescribing behaviours which result in inappropriate antibiotic prescribing practices, which existing evidence suggests are likely to contribute to AMR. The development and implementation of behavioural interventions based on evidence are one way in which the emerging and developing public health problem of AMR can be addressed.

## Abbreviations

AMR: Antimicrobial Resistance
AMS: Antimicrobial Stewardship
CARS Advisory Group: Control of Antimicrobial Resistance Scotland Advisory Group
HPS: Health Protection Scotland
